# Adoption of artificial intelligence in the HRM function within the South African telecommunication sector: a quantitative analysis using structural equation modelling

**DOI:** 10.3389/frai.2026.1807550

**Published:** 2026-05-05

**Authors:** William Makumbe, Lungile Ntsizwane, Ntseliseng Khumalo

**Affiliations:** Business School, Northwest University, Potchefstroom, South Africa

**Keywords:** artificial intelligence, HRM, South Africa, telecoms, SEM

## Abstract

**Introduction:**

The rapid emergence of the digital workplace has accelerated the integration of artificial intelligence (AI) into human resource management (HRM). However, empirical research on the determinants of AI adoption in organisational contexts remains limited, particularly in emerging economies. This study examines the factors influencing AI adoption in HRM within the South African telecommunications sector.

**Methods:**

A quantitative approach was adopted, with data collected from 500 HR managers selected systematically. Structural equation modelling (SEM) was used to test relationships between performance expectancy, effort expectancy, facilitating conditions, attitudes, behavioural intention, and actual AI adoption.

**Results and discussion:**

Performance expectancy and effort expectancy significantly and positively influenced attitudes towards AI adoption. Facilitating conditions and attitudes were significant predictors of behavioural intention to use AI technologies. Behavioural intention, in turn, had a strong positive effect on actual AI adoption in the workplace. The findings extend existing literature on technology adoption in HRM, particularly in emerging economy contexts. They underscore the importance of perceived usefulness, ease of use, and organisational support in driving AI adoption. For practitioners, the results highlight the need to strengthen enabling conditions and foster positive attitudes to support the successful implementation of AI-driven HR practices.

## Introduction and background

The emergence of the Fourth Industrial Revolution has propelled the digitalisation of workplaces. The rapid advancement of information technology (IT) in the workplace has enabled the migration of human resources management (HRM) into the digital domain (Zhou et al., 2021). Digital tools such as the Internet of Things, robotics, and artificial intelligence have become a priority for business managers (Chowdhury et al., 2023). These IT innovations are playing a critical role in the transformation of HRM (Hmoud, 2021). The transformative nature of artificial intelligence in the workplace (Kshetri, 2020) has made its use and adoption in HRM a prominent topic. As a result, there has been a proliferation of research work on artificial intelligence in the HRM domain (see Budhwar et al., 2022; Chowdhury et al., 2023; Gryncewicz et al., 2023; Kshetri, 2020; Malik et al., 2020, 2023; Strohmeier and Piazza, 2015; Aldulaimi et al., 2020; Chatterjee and Bhattacharjee, 2020; Hmoud, 2021; Hossin et al., 2021; Pereira et al., 2023; Tambe et al., 2019; Tewari and Pant, 2020; Vrontis et al., 2022). This growing body of evidence confirms that artificial intelligence has become a critical component for the effective performance of the HRM function. However, the majority of these studies are based in the Global North; hence their findings cannot be directly applied in the Global South, particularly in sub-Saharan Africa, where unique business cultural environments exist.

Previous research shows that the majority of studies on artificial intelligence in the HRM domain have largely been qualitative (see Alsheibani et al., 2020; Budhwar et al., 2022; Chowdhury et al., 2023; Ekuma, 2024; Gryncewicz et al., 2023; Malik et al., 2020, 2023; Pereira et al., 2023; Upadhyay and Khandelwal, 2018; Vrontis et al., 2022). These studies have focused on the value of AI in HRM (Chowdhury et al., 2023), AI-enabled strategic framework (Malik et al., 2023), benefits of AI in HRM (Gryncewicz et al., 2023), AI and recruitment (Upadhyay and Khandelwal, 2018), challenges and opportunities of AI for International HRM (Budhwar et al., 2022), and the role of AI in human resource development (Ekuma, 2024). This implies that there is a growing need for quantitative studies on AI in HRM. Utilising the unified theory of acceptance and use of technology (UTAUT), this study quantitatively investigates managers’ perceptions of adopting AI in the HRM function in the South African telecommunication industry, which is one of the most diversified sectors in Africa.

South Africa is ranked 59th out of 132 economies on the Global Innovation Index (World Intellectual Property Organization, 2022). Unsurprisingly, the country’s telecoms sector boasts one of the most advanced information technology infrastructures on the continent (www.budde.com.au, 20/07/2024). This diverse and competitive sector includes major conglomerates such as Telecom, Liquid Intelligent Technologies, MTN, Vodacom, and Eskom. By their nature, these organisations are high-tech companies, and AI plays a crucial role in driving their operations.

Investigating manager’s perceptions towards the adoption of AI in this sector is therefore useful in shaping the HR strategy (Kshetri, 2020).

A study of this nature yields significant contributions to the field of HR management. First, it addresses the recent calls for increased research on the role of artificial intelligence in HRM (Malik et al., 2020, 2023; Vrontis et al., 2022). Second, given that the majority of research on artificial intelligence is confined to the Global North, this study contributes to the literature from the Global South perspective. From a practical standpoint, the results of this study can offer important insights for effective decision-making amongst HR practitioners in the telecommunication sector.

This study is organised as follows: the next section reviews the literature, followed by the development and discussion of the results.

## Literature review

### Artificial intelligence in HRM

According to Tambe et al. (2019), artificial intelligence refers to a broad class of technologies that allow a computer to perform tasks that normally require human cognition, including adaptive decision-making. Generally, AI has been used in fields such as manufacturing, sports, healthcare, banking, and education. Specifically, it has had a positive impact on functions such as marketing, production, and human resources management (HRM). AI integration in HRM has become increasingly popular due to its potential to shift the HRM paradigm to a new era. The importance of AI is observable in four main areas, namely decision-making, recruitment and selection, training and development, performance management, and employee engagement and retention. A brief discussion of each of these is presented below.

#### Strategic decision-making

AI can enhance decision-making processes by providing the required knowledge to executives at the appropriate time (Reina and Scarozza, 2021). Services that utilize various AI tools can benefit organisations in various ways, such as enabling accurate and timely decision-making, minimising processing time and data errors, increasing service flexibility and accuracy, and enhancing problem-solving capabilities, as well as generating unique sets of outcomes and knowledge. AI can help identify the competencies an organisation lacks, forecast which employees are not likely to promoted, and pinpoint which workers may leave organisation. In HRM, AI performs tasks efficiently than HR professionals, particularly when it comes to repetitive tasks such as collecting and updating employee data (Korteling et al., 2021).

#### Recruitment and selection

Recruitment and selection are highly speculative procedures where AI might be used provocatively. According to Markauskaite et al. (2022), incorporating science, AI, and analytics into the recruitment process can help mitigate biases and enhance decision-making in candidate selection. HRM aims at getting the best employees, and finding them through a clear understanding and utilisation of AI should be a priority. The majority of companies use applicant tracking systems (ATSs), which provide recruiters with an overview of their job postings and help them manage applicant data effectively (Allal-Chérif et al., 2021). By leveraging AI technologies, companies can simplify the recruitment and selection processes, offering capabilities such as efficient carryover of data from resumes, sorting of efficient candidate data, providing the organisation with a platform capable of secure data access, outcome forecasting, and appointment or selection processes, and clarifying preferences when addressing concerns or more complicated issues (FraiJ and László, 2021).

#### Training and development

AI can be easily integrated into various training and development programmes. Nazaretsky et al. (2022) indicated that educators in the field of corporate training can develop systems to tailor training programmes to meet the specific needs of professionals across various positions. AI and machine learning enable computer systems to independently construct algorithms and statistical models without human intervention. This capability allows computers to draw conclusions, make potential decisions and improve the training environment and overall efficiency within the workplace (Verma et al., 2022). AI can be used before, during, or after the training process for both design and delivery, as well as for assessment purposes. Furthermore, AI can support the design of training by acquiring knowledge, skills, and behaviour assessments of participants and identifying their needs. As a result, AI personalises learning pathways and experiences and helps develop optimised content.

#### Performance management

In performance management, performance measurement is the first level, providing real-time feedback through monitoring, goal tracking, and behaviour assessments (Atmaja, 2024; Awan et al., 2020; Al-Jedaia and Mehrez, 2020). Emerging AI and big data technologies facilitate the establishment of performance standards, maintain continuous visibility into the recruitment process, and allow the monitoring and direct provision of real-time feedback (Allal-Chérif et al., 2021). This approach fosters a more distanced form of communication between employees and managers, helping teams continuously adjust and aim for high-performance standards. In organisations creating real-time feedback systems, both managers and employees benefit because real-time feedback leads to higher job satisfaction and productivity (Burnett and Lisk, 2021; Martin et al., 2022; Davidescu et al., 2020). The most common use of AI in the realm of HR appears to be in predicting and selecting candidates who are most likely to perform and less likely to leave, quite often with a specific focus on areas such as education and leadership (Ore and Sposato, 2022; Alam et al., 2020). As a result, AI can be used to help identify internal talent with the potential to fill upcoming or currently vacant positions. However, other research suggests that this is less likely, with some arguing that AI is more focused on developing employee competencies rather than filling high-level positions (Allal-Chérif et al., 2021; Pillai and Sivathanu, 2020; Ore and Sposato, 2022).

#### Employee engagement and retention

Employee engagement and satisfaction play an important role in sustaining a secure internal talent base that can adapt to and drive organisational change (Malik and Garg, 2020). Engagement can occur in various ways, including the use of employee surveys to gather feedback. Surveys are a common practice amongst organisations, particularly for assessing employee feelings and satisfaction or evaluating the work environment. Employee surveys have been enhanced by advances in technology, which have led to the application of expert systems and machine learning to select employees for training and to predict employee turnover (Burnett and Lisk, 2021; Ho and Kuvaas, 2020). These systems can also be applied to more general characteristics, such as organisational culture. The more employee surveys conducted, the more insights gained about the organisation. Some surveys use AI to instantly analyse large amounts of data. Organisational operations, culture, and other variables are then compared against departmental, unit, or organisation results. In addition to providing insights into employee engagement, it can also be used to assess the organisation’s pay strategy (Zhang and Zhang, 2022).

#### Workforce planning and succession management

Workforce planning is the process that matches internal and external resources with established organisational goals (Kehoe and Han, 2020). For the most strategic aspect of workforce planning, predictive workforce analytics are increasingly used (Konovalova et al., 2021). These predictive workforce analytics start with historical personnel data and try to forecast workforce needs in the future (Cho et al., 2023; Arora et al.,2021; Kakulapati et al.,2020; Gurusinghe et al., 2021). If employees are the most important organisational resource, accurate workforce forecasts can lead to an improved allocation of employees. One advantage of using historical personnel data as a predictor is the ability to account for past trends and events in the workforce forecast. AI can link replacement planning closely to business needs. Succession planning is about identifying and preparing future leaders within the ranks of the organisation. Succession planning predictions focus on identifying and nurturing the new leaders of an organisation (Siambi, 2022). These predictions can be based on historical data that focuses on objective evidence of current employee performance, employee competencies, and employee potential for growth and improvement.

## Theoretical framework

### Unified theory of acceptance and use of technology (UTAUT)

Many models and theories have been applied to examine the acceptance and use of information systems (Ayaz and Yanartaş, 2020). The UTAUT, which forms the basis of this investigation, has been applied in different contexts and has proven to be a useful tool for measuring the intention and the actual adoption of information technologies (Ronaghi and Forouharfar, 2020) and the probability of success of new technology (Ayaz and Yanartaş, 2020). The UTATUT was developed by redefining representative technology acceptance theories such as the Theory of Reasoned Action (TRA), the Technology Acceptance Model (TAM), and the Theory of Planned Behaviour (TPB) into an integrated perspective (Sohn and Kwon, 2020). The main constructs in the UTAUT are performance expectancy, effort expectancy, social influence, and facilitating conditions. These factors are considered crucial to the adoption of technology.

Owing to its robustness and its ability to explain 70% of technology use, the UTAUT underpins this study. The four constructs of UTATUT are particularly relevant for examining AI adoption in HRM because the successful integration of AI technologies—such as automated recruitment systems, predictive analytics, and AI-driven employee support tools—depends largely on HR professionals’ perceptions of the usefulness, ease of use, and organisational support associated with these technologies. Performance expectancy explains the extent to which HR practitioners believe that AI can enhance efficiency, improve decision-making, and optimise talent management processes. Effort expectancy captures the perceived ease of learning and using AI-driven HR systems, which is critical in determining user acceptance. Social influence reflects the role of organisational leadership, colleagues, and industry pressures in shaping attitudes towards AI adoption, whilst facilitating conditions emphasise the importance of organisational infrastructure, training, and technical support in enabling the effective use of AI technologies. Given its strong predictive power and extensive empirical validation in studies of emerging technologies, the UTAUT framework provides an appropriate and comprehensive foundation for analysing the determinants of AI adoption within HRM contexts.

### Hypothesis development

#### Performance expectancy and attitudes towards AI

Performance expectancy is defined as the extent to that the user believes that using the new system would enhance job performance (Chatterjee and Bhattacharjee, 2020). In support of this, Ronaghi and Forouharfar (2020) opined that performance expectancy refers to the degree to which an individual thinks that technology usage could facilitate the attainment of better performance in given tasks. Performance expectancy is construed to be similar to the perceived usefulness variable of the technology acceptance model (Chatterjee and Bhattacharjee, 2020). In the context of artificial intelligence adoption in human resource management (HRM), performance expectancy is likely to positively influence attitudes towards AI when HR professionals perceive that AI tools can significantly improve the effectiveness and efficiency of HR functions. For example, AI applications can automate routine administrative tasks, improve recruitment accuracy through data-driven candidate screening, enhance workforce analytics, and support more informed decision-making in talent management. When HR practitioners recognise these potential benefits, they are more likely to develop favourable attitudes towards the adoption of AI technologies. In other words, the stronger the perception that AI will improve job performance and organisational outcomes, the more positive the attitude towards adopting such technologies. Previous research confirms that performance expectancy has a positive impact on the attitude towards the use of technology (see Ayaz and Yanartaş, 2020; Chatterjee and Bhattacharjee, 2020; Ronaghi and Forouharfar, 2020). It can thus be hypothesised that:

*H1:* Performance expectancy positively influences the attitude towards the use of artificial intelligence.

#### Effort expectancy and attitudes towards AI

Effort expectancy is defined as the extent of simplicity regarding the use of a new system (Chatterjee and Bhattacharjee, 2020). In other words, it defines how convenient (Ronaghi and Forouharfar, 2020) it is to use new technologies in an organisation. Other authors such as Ayaz and Yanartaş (2020) stated that effort expectancy is a measurement of a system design, ease of use, and flexibility. It, therefore, implies that technologies that are perceived as easy to learn and operate are more likely to be accepted by users. In the case of AI adoption in HRM, if HR professionals perceive AI systems—such as AI-powered recruitment platforms, chatbots, or analytics tools—as user-friendly and not overly complex, they are more likely to develop a positive attitude towards their adoption. Conversely, if AI systems are perceived as complicated or requiring extensive technical expertise, employees may resist their use. Therefore, when AI technologies are designed with intuitive interfaces, adequate training support, and seamless integration into existing HR systems, they reduce perceived effort and increase user confidence, thereby fostering more favourable attitudes towards AI adoption. Prior studies confirmed the predictive power of effort expectancy on attitudes (see Ayaz and Yanartaş, 2020; Gupta and Arora, 2020; Sohn and Kwon, 2020).

*H2:* Effort expectancy positively influences the attitude towards artificial intelligence usage.

#### Facilitating conditions and behavioural intention

Facilitating conditions are defined as the extent to which an individual believes that the conducive technical and allied infrastructure is effectively available to support the usage of the new system (Chatterjee and Bhattacharjee, 2020). The existence of organisational infrastructure to support the usage of the new system or technology is likely to influence the behavioural intention of the users (Ronaghi and Forouharfar, 2020). In the context of artificial intelligence (AI) adoption in human resource management (HRM), facilitating conditions play a crucial role in shaping individuals’ willingness and intention to adopt AI systems. When employees perceive that their organisation has adequate technological infrastructure, training programmes, technical support, and clear policies to support the implementation of AI technologies, they are more likely to develop a strong behavioural intention to use such systems. For example, the availability of reliable IT systems, access to AI tools, and continuous training can reduce uncertainty and increase users’ confidence in their ability to interact with AI-driven HR applications. In addition, organisational support signals management commitment to digital transformation, which can further motivate employees to embrace AI technologies in their daily HR practices. Previous studies have supported the positive impact of facilitating conditions on the behavioural intention of users (see Ayaz and Yanartaş, 2020; Gupta and Arora, 2020). It can therefore be hypothesised that:

*H3:* Facilitating conditions positively influence the behavioural intention towards artificial intelligence usage.

#### Attitudes towards AI and behavioural intention

Feelings are involved in the performance of a target behaviour. According to Makumbe and Mutsikiwa (2021), attitude towards the use of technology refers to the extent to which a user experiences positive feelings about using such technology. Users of artificial intelligence can either develop negative or positive feelings about engaging in a certain behaviour towards its use. Their feelings depend on how easy and useful the technology is for performing the assigned tasks. Attitudes, hence, act as a strong mediator in influencing behavioural intention (Chatterjee and Bhattacharjee, 2020). It can thus be proposed that:

*H4:* Attitude has a strongly significant impact on the behavioural intention towards the adoption of artificial intelligence.

#### Behavioural intention and the adoption of AI

According to Punnoose (2012), behavioural intention refers to a cognitive representation of a person’s readiness to perform a given behaviour. Managers might be ready or not ready to use artificial intelligence, depending on their attitude towards the technology. This implies that if the attitude towards AI is positive, this can result in managers adopting AI in the workplace. It can therefore be proposed that:

*H5:* Behavioural intention positively influences the actual adoption of AI.

**Figure 1:**
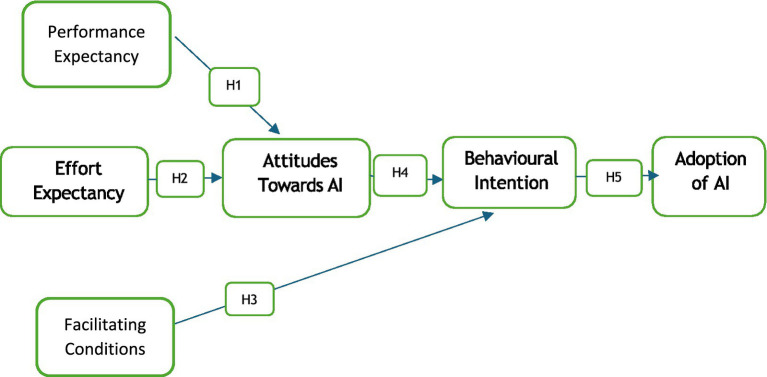
Research model. Source: own creation.

In line with the discussion above, this investigation proposes the research model in Figure 1.

## Research methods

### Participants

There search participants comprised 500 managersan demployeessystematically selected from major telecommunication service providers in South Africa. Data were collected using a structured questionnaire through Google Forms. A questionnaire link was created and sent to respondents via WhatsApp and email.

### Research instrument measurement scales

Prior research work items were used to measure the research constructs. The measurement items for effort expectancy, facilitating conditions, attitude, behavioural intention, and adoption were adapted from Chatterjee and Bhattacharjee (2020). Performance expectancy and adoption were measured using items adapted from the work of Ratten (2015).

### Reliability and validity

Data reliability and validity were assessed using indicator reliability, Cronbach’s alpha, average variance extracted (AVE), composite reliability (CR), and discriminant validity.

#### Indicator reliability

Reflective indicator loadings greater than 0.5 show that the item is a good measure of a latent construct (see Hulland, 1999). Accordingly, all indicator loadings exceeded 0.5 (see Table 1).

**Table 1:** Questionnaire items, factor loadings, means, standard deviation Cronbach’s values, average variance extracted, and composite reliability.

		Factor loading	Alpha	AVE	CR
Code	Performance expectancy		0.80	0.55	0.83
PE1	AI-powered HRM will enhance the efficiency of the organisation	0.65			
PE2	HR activities prepared by AI technology are useful	0.73			
PE3	Using AI-powered HR technology brings convenience.	0.74			
PE4	Smart HRM content can be prepared using AI technology	0.84			
	Effort expectancy		0.82	0.62	0.86
EE1	HR individualised content can be prepared using AI technology.	0.72			
EE2	AI-powered HRM technology is easy to learn	0.73			
EE3	I need to put a lot of effort into learning HR AI technology	0.82			
EE4	I can have my query answered quickly using AI- HR chatbot technology	0.88			
	Facilitating conditions		0.79	0.63	0.87
FC1	This organisation encourages its staff to use modern technology	0.78			
FC2	This organisation has all the necessary resources to use AI technology for smart HR content creation	0.87			
FC3	This organisation sponsors any AI-HR-related learning opportunity	0.77			
FC4	We have all the required resources to develop AI-powered HR-based smart content	0.74			
	Attitude		0.83	0.57	0.84
AT1	AI-powered HR technology is pleasant.	0.81			
AT2	Using AI technology is a good idea	0.75			
AT3	Using AI HR technology makes me feel comfortable	0.83			
AT4	I feel good when using AI-powered HR technologies	0.62			
	Behavioural intention		0.86	0.60	0.85
BI1	I prefer using AI-powered HR technology when doing my job	0.76			
BI2	I am willing to use AI-powered HR technology to develop smart content	0.85			
BI3	I shall recommend all managers in the telecoms sector explore AI powered HR technology.	0.72			
BI4	I intend to continue using AI-powered HR technology in the foreseeable future	0.77			
	Adoption of AI in HRM		0.72	0.59	0.81
AD1	I plan to adopt AI-powered HR technologies in this organisation	0.73			
AD2	I am willing to use AI-powered HR technologies	0.78			
AD3	I will definitely use AI-powered HR technologies in this organisation	0.81			

#### Internal consistency reliability

Composite reliability (CR) and Cronbach’s alpha (*α*) can be used to assess internal consistency reliability. Gefen et al. (2000) state that a CR value of at least 0.7 indicates adequate internal consistency reliability. Hair et al. (2017) suggested that Cronbach alpha (α) values between 0.60 and 0.70 are widely considered desirable in research to indicate internal consistency reliability. As shown in Table 1, all constructs met the Cronbach’s alpha and composite reliability threshold values.

#### Convergent reliability

Convergent reliability is the extent to which a measure correlates positively with alternative measures of the same construct (Hair et al., 2017). Convergent reliability is assessed using the average variance extracted (AVE). The AVE should be greater than 0.5 (see Bagozzi, 1986; Hair et al., 2016). The AVE for all constructs in this study was greater than 0.5 (see Table 1); thus, the measurement scales showed good convergent reliability.

### Discriminant validity

Discriminant validity was assessed using the Fornell–Larcker criterion (see Hair et al., 2016). The Fornell–Lacker criterion compares the square root of the AVE values with the latent variable correlations with other constructs. The square root of the AVE of each construct should be higher than the correlation with any other construct (Hair et al., 2016). The square root of the AVE of each latent variable is shown diagonally in bold in Table 2, along with the correlations of the latent variable with other latent variables. Table 2 indicates that the square root of the AVE of each latent variable is indeed higher than any correlation with any other latent variable. Thus, the measurement instrument satisfied discriminant validity.

**Table 2:** Discriminant validity.

Variable	Performance expectancy	Effort expectancy	Facilitating conditions	Attitude	Behavioural intention	Adoption of AI
Performance expectancy	0.74					
Effort expectancy	0.70	0.79				
Facilitating conditions	0.69	0.73	**0.78**			
Attitude	0.68	0.71	**0.76**	**0.75**		
Behavioural intention	0.65	0.70	**0.75**	**0.70**	**0.77**	
Adoption of AI	0.66	0.72	0.72	**0.71**	**0.75**	**0.76**

The discriminant validity values are shown diagonally in bold.

### Confirmatory factor analysis

The suitability of data for factor analysis was assessed using the Kaiser–Meyer–Olkin and Bartlett’s test of Sphericity. The KMO value was 0.86, above the minimum recommended value of 0.5 (see Hair et al., 2016). In addition, Bartlett’s test of Sphericity was significant at a *p*-value of 0.000, rendering the sample adequate for factor analysis.

In assessing the model, the researchers used the following model fit indices: CMIN, IFI, CFI, and the RMSEA. After running the confirmatory factor analysis for the model, the results indicated that the model was good because it produced results that were within acceptable limits (see Hair et al., 2016). The model fit indices obtained from the confirmatory factor analysis are CMIN = 1.56; *p* = 0.000; IFI = 0.91; CFI = 0.91; RMSEA = 0.07.

### Structural equation modelling

**Figure 2:**
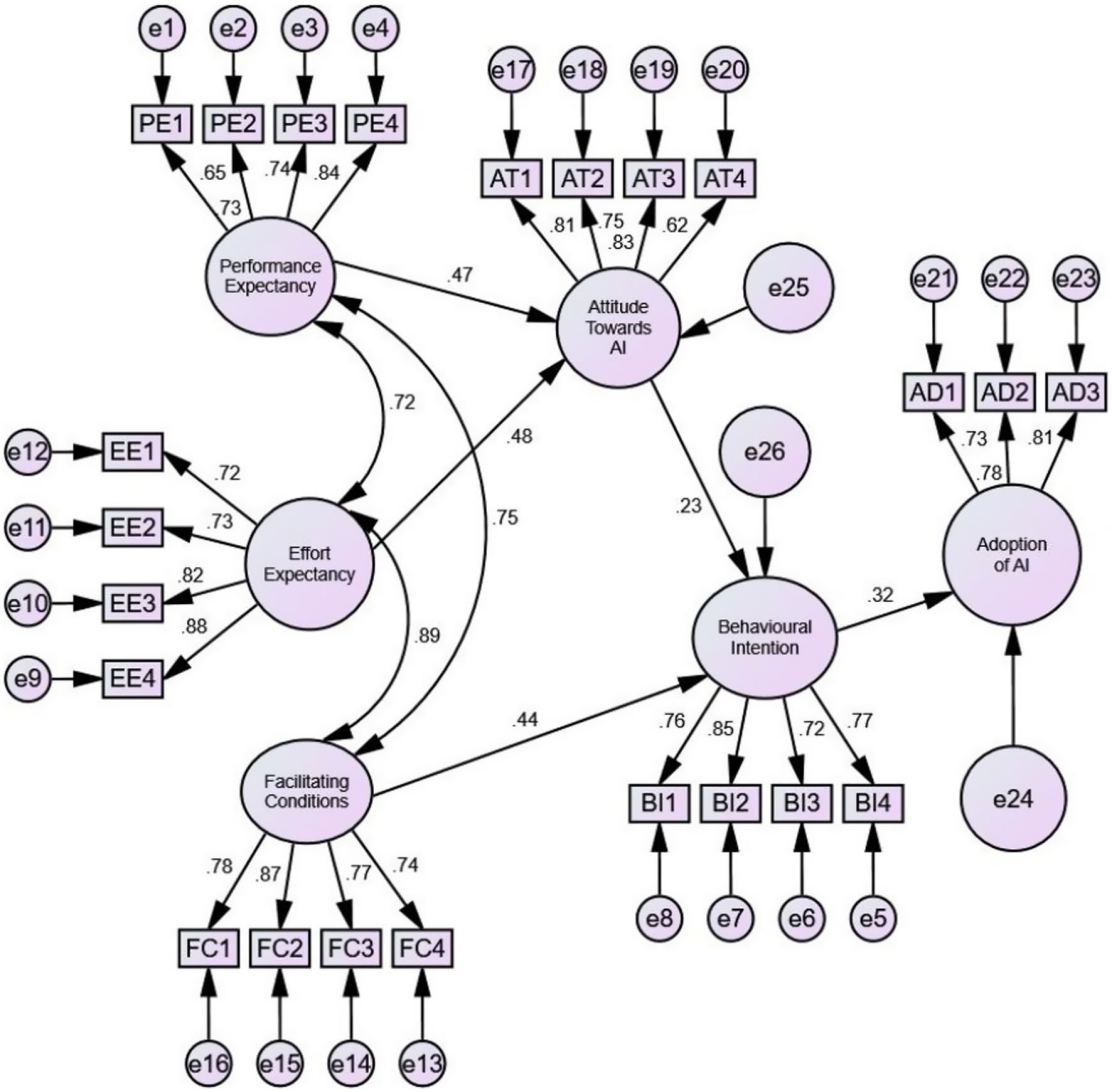
SEM graphic output. Source: SPSS output.

Figure 2 shows the results of the SEM analysis conducted using Amos version 26. Table 3 indicates that all model fit values were within the acceptable range.

**Table 3:** Model fit indices.

Model fit index	Scores	Recommended value
CMIN/Df	1.56	1 < CMIN/Df < 5
Root mean square error of 0.07 approximation (RMSEA)	0.07	<0.08
Incremental fit index (IFI)	0.91	< 1
Comparative fit index (CFI)	0.91	>0.9

### Direct effects

As indicated in Table 4, performance expectancy and effort expectancy significantly impacted the intention to adopt AI (*β* = 0.47, *p* = 0.00; β = 0.48, *p* = 0.00, respectively). Hence, *H1* and *H2* were accepted. Furthermore, facilitating conditions and attitude had a significant impact on behavioural intention to adopt AI (β = 0.44, *p* = 0.01; β = 0.23, *p* = 0.00 respectively). Hence, H3 and H4 were accepted. Finally, behavioural intention had a profound positive impact on the actual adoption of AI (β = 0.32, *p* = 0.01; *β* = 0.23, *p* = 0.00).

**Table 4:** Path coefficients and probability values.

Hypothesis	Path	Path	*p*-value	Decision
H1	Performance expectancy → Attitude	0.47	0.02	Accepted
H2	Effort expectancy → Attitude	0.48	0.00	Accepted
H3	Facilitating conditions → Behavioural intention	0.44	0.01	Accepted
H4	^Attitude^ → ^Behavioural Intention^	0.23	0.00	Accepted
H5	^Behavioural intention^ → Adoption of AI	0.32	0.00	Accepted

## Discussion of findings

This study investigated the attitude of South African Telecoms sector HR managers towards the adoption of artificial intelligence in the workplace. There are several viewpoints to be considered. First, as hypothesised, performance expectancy was positively related to the attitude variable (β = 0.47, *p* = 0.00). This finding resonates with prior studies (see Ayaz and Yanartaş, 2020). In the context of this study, the result implies that artificial intelligence has proven to be useful in the workplace, hence managers have a positive feeling towards its use. AI is useful in such areas as the pre-selection of employees (Hmoud, 2021), turnover prediction (Strohmeier and Piazza, 2015), revenue generation, and cost management (Alsheibani et al., 2020). All these benefits are not just important to the HR division but to the entire organisation. Second. Effort expectancy had a positive impact on the attitude variable (*β* = 0.48, *p* = 0.00). This finding corroborates the work of Gupta and Arora (2020). Interactivity complexity of AI is relatively easy, hence less effort is required to use AI-enabled systems. AI-enabled applications such as chatbots, intelligent search engines, and smart applicant tracking systems are relatively easy to use. The use of such AI-based systems in e-recruiting, training, and sentiment analysis is associated with increased accuracy of results and decreased effort (Strohmeier and Piazza, 2015). As a result, system users can easily develop a positive attitude towards their use.

Third, in line with prior studies (see Chatterjee and Bhattacharjee, 2020), facilitating conditions were found to have a positive impact on the behavioural intention variable (*β* = 0.44, *p* = 0.01). This finding confirms the existence of supportive infrastructure for AI adoption in the South African telecoms sector. South Africa boasts of a well-developed information technology landscape, with the country ranked at position 67 out of 132 countries on the Information and Communication Technology (ICT) Access Index (see Global Innovation Index, 2023). The penetration of ICT is mainly driven by supportive government policies such as the South Africa Connect policy (Mwapwele et al., 2019). Hence, the availability of ICT infrastructure, such as hardware, software, and internet connectivity, facilitates the intention to adopt AI in the HR function in many telecoms organisations in South Africa.

Fourth. The attitude variable had a positive impact on behavioural intention (β = 0.23, *p* = 0.01). This finding agrees with several technology adoption studies (see Al-Emran et al., 2020; Ansong-Gyimah, 2020; Buabeng-Andoh, 2021; Rafique et al., 2020). As Teo (2019) noted, the attitude of employees shapes their behavioural intentions. Hence, in the context of this study, South African HR managers are exhibiting positive attitudes towards AI use and adoption, and this, in turn, is influencing their behavioural intention. Last, in line with prior studies such as Chatterjee and Bhattacharjee (2020) behavioural intention was found to be positively related to the actual adoption of AI (β = 0.32, *p* = 0.00). As behavioural intention is a key predictor of actual adoption, this finding is a key indicator of the perception of South African HR managers towards the adoption and use of AI-enabled systems. South African HR managers are ready and willing to use artificial intelligence.

### Theoretical implications

This study is theoretically significant in several ways. First, in line with previous findings (see Ayaz and Yanartaş, 2020; Chatterjee and Bhattacharjee, 2020; Gupta and Arora, 2020), this investigation confirmed that the attributes of UTAUT positively influence users’ attitudes and behavioural intentions regarding the acceptance of technology. This implies that this study makes an important theoretical contribution by extending the applicability of the Unified Theory of Acceptance and Use of Technology (UTAUT) to the emerging context of artificial intelligence adoption in human resource management (HRM). Although the UTAUT framework has been widely used to explain the acceptance of information technologies, limited research has examined its relevance to AI-driven HR practices. By demonstrating that performance expectancy, effort expectancy, social influence, and facilitating conditions positively influence attitudes towards AI adoption, this study provides empirical support for the continued explanatory power of UTAUT in contemporary digital organisational environments. Furthermore, the findings contribute to theory by highlighting the importance of attitudinal mechanisms in shaping AI adoption behaviour, suggesting that attitudes may play a critical role in linking technology-related perceptions to adoption decisions within HR functions. In doing so, the study advances the theoretical integration between technology adoption theories and the growing literature on digital and AI-enabled HRM, thereby offering a refined understanding of the behavioural and organisational factors that underpin the successful implementation of AI technologies in HR practices. Second, this research extends the limited body of evidence on the role of artificial intelligence in HRM. This is especially true in Africa, where technology-related research work is required in different sectors. Furthermore, artificial intelligence research has largely been qualitative (see Alsheibani et al., 2020; Budhwar et al., 2022; Chowdhury et al., 2023). This research offers an improved understanding of the impact of artificial intelligence in HRM from a quantitative perspective.

### Practical implications

This study offers important practical insights to organisational stakeholders such as technology system developers, HR managers, employees, and the government. This study’s findings confirm that artificial intelligence attributes such as efficiency and ease of use (effort expectancy) are crucial in influencing the attitudes of users towards its adoption. This is an important insight for technology systems developers in general. To enhance the market acceptability of their products, technology developers must ensure that software inbuilt mechanisms can meet the performance needs of clients. This implies that by using technology, clients must be able to attain their task targets within the stipulated time. This is possible only if technology downtime and errors are limited or non-existent. Hence, technology systems developers must always aim for customer delight by matching or exceeding their expectations.

In the context of this research, technology systems developers must ensure that artificial intelligence software deployed in HR is effective in such areas as recruitment, selection, turnover prediction, and productivity analysis.

As facilitating conditions were found to influence behavioural intention positively, the telecom executives must support the development of technology infrastructure at the workplace. Telecom executives in general must allocate budgetary resources for the acquisition of appropriate technology. This implies the provision of the financial resources required to support the acquisition of hardware and software infrastructure, network infrastructure, and security infrastructure. Specifically, HR managers must also train their employees to function effectively in a digitalised environment. An investment in information technology that is not supported by training cannot lead to the desired results.

The findings of this research are essential to African governments as they are responsible for creating and facilitating the conditions for investment in information and communication technologies. In Africa, the adoption of technology has been sluggish owing to a weak technology landscape (Dutta et al., 2022). This does not bode well for a continent that is aiming to compete on global markets. African governments must, therefore, strive to create a conducive atmosphere to attract direct technology investment from both domestic and international investors. The existence of multiple players in the information technology sector creates healthy competition, which will ultimately benefit end users. Furthermore, the African governments must provide the requisite resources to support the development of technology infrastructure. Not only that, but also through higher education institutions, African governments must spearhead the training of information technology specialists who can function effectively in a digitalised environment. The idea here is to develop both system developers and users in Africa.

Study findings are equally important to employees as they are to managers and the government. Employees must realise that the workplace is now technology-driven. This calls for their attitudinal change towards the use of technology in the workplace. Personal development is now more paramount than ever. Hence, information and communication technology training should be prioritised from an employee perspective.

## Conclusion

This study confirmed that users of artificial intelligence in the HRM function have a positive attitude towards its adoption. This is mainly driven by the efficiency and ease of use attributes of the software. Hence, technology system developers must strive to create technologies with functional attributes that facilitate exceptional performance in different business areas. Further, this study confirmed the importance of facilitating conditions as a driver of influencing the behavioural intention of users towards the acceptance of artificial intelligence in HR. This implies that governments and managers must play a supportive role in ensuring the creation of an enabling environment for the development of technology infrastructure.

### Limitations and future research direction

This study addresses an important concept in business management and has improved our understanding of technology use and adoption in business. However, the study was done in the South African setting, with a sample of 500 managers. The sample size of 500 managers might represent a small fraction; hence, scholars may need to test our conceptual model in a different context with a larger sample. The items used to measure the adoption of artificial intelligence reflected mostly positive dispositions, such as “enhance,” “convenience,” “pleasant,” “comfortable,” and “easy.” It may be necessary to discuss or propose, for future research, the evaluation of negative attitudes about the use of artificial intelligence in human resource management. Despite these limitations, this study provided practical empirical evidence demonstrating the relationship amongst the variables investigated in this study.

## Data Availability

The raw data supporting the conclusions of this article will be made available by the authors, without undue reservation.
